# Comparative Analysis of Long-Term Measles Immune Response After Natural Infection and Routine Vaccination in China

**DOI:** 10.3390/vaccines13060555

**Published:** 2025-05-23

**Authors:** Sihong Zhao, Qianli Wang, Juan Yang, Qiaohong Liao, Juanjuan Zhang, Xiaoyu Zhou, Jiaxin Zhou, Zeyao Zhao, Yuxia Liang, Junteng Luo, Jingting Cai, Yanpeng Wu, Wei Wang, Hongjie Yu

**Affiliations:** 1 Key Laboratory of Public Health Safety, Ministry of Education, School of Public Health, Fudan University, Shanghai 200032, China; shzhao22@m.fudan.edu.cn (S.Z.); yangjuan@fudan.edu.cn (J.Y.); liaoqiaohong1983@163.com (Q.L.); zhangjuan@fudan.edu.cn (J.Z.); jxzhoufd@163.com (J.Z.); 21111020047@m.fudan.edu.cn (Z.Z.); scudxliangyuxia@163.com (Y.L.); 24111020054@m.fudan.edu.cn (J.L.); jtcai20@fudan.edu.cn (J.C.); 2 Shanghai Institute of Infectious Disease and Biosecurity, Fudan University, Shanghai 200032, China; wang_ql@fudan.edu.cn (Q.W.); 22211020043@m.fudan.edu.cn (X.Z.); 21111020024@m.fudan.edu.cn (Y.W.); 3 Department of Infectious Diseases, Huashan Hospital, Fudan University, Shanghai 200032, China

**Keywords:** measles, vaccination, natural infection, antibody dynamics

## Abstract

**Background:** Given the significant impact of population immunity on the measles epidemic, understanding immunity differences among populations with varying immunity backgrounds is necessary for identifying immunity gaps and informing vaccination policies. In this study, we aimed to determine the distinct dynamics of vaccine-induced and naturally acquired antibodies, with specific focus on difference in vaccine-induced antibody responses across different birth cohorts. **Methods:** Based on two cohorts and one cross-sectional study conducted in Anhua County, Hunan Province, China, serum samples from children who followed China’s routine measles vaccination schedule (i.e., two-dose schedule at 8/18 months) and adults who acquired immunity through natural infection were tested for measles IgG antibodies using an enzyme-linked immunosorbent assay. The generalized additive mixed model and a mechanistic model were employed to describe antibody dynamics following vaccination and infections. Wavelet analysis was used to investigate the temporal relationship between the measles epidemic and long-term antibody levels after natural infection. **Results:** A total of 408 children (0–12 years) and 222 adults (54–84 years) were included in the present study. Vaccine-induced antibody levels following 8 m/18 m vaccination were estimated to fall below the protective threshold of 200 mIU/mL by age of 15.8, whereas antibody levels following infections remained high. The decay rate of vaccine-induced antibodies was estimated at 3.0 × 10^−3^ log-log mIU/mL per year, whereas naturally acquired measles antibodies persisted lifelong with a significantly lower decay rate of 2.30 × 10^−5^ log-log mIU/mL per year. Moreover, vaccine-induced antibody levels in children born after 2010—a period of low measles incidence—declined more rapidly (duration of protective immunity: 12.5 years), compared to those born before 2010. **Discussion:** Our findings revealed immunity heterogeneity among individuals with difference measles immunity backgrounds. In particular, the birth-cohort specific differences in vaccine-induced immunity highlighted the key role of young generations born in settings with low measles incidence in contributing to population immunity gaps. This underlines that greater attention should be given to this group in future catch-up vaccination efforts.

## 1. Introduction

Measles is a highly contagious but vaccine-preventable disease [1]. In China, the measles-containing vaccine (MCV) was introduced in the 1960s and incorporated into the Expanded Program on Immunization in 1978, with a routine two-dose schedule administered at 8 and 18 months of age. Nationwide implementation of this routine two-dose vaccination, along with intermittent supplementary immunization activities between 2003 and 2010, has substantially reduced measles morbidity and mortality compared to the pre-vaccine era (morbidity: 26 versus 6063 cases per million; mortality: 0 versus 99 deaths per million) [2]. Since then, due to continuous virus circulation and the nationwide implementation of routine and supplementary immunization activity (SIA) vaccination, the immunological background of the Chinese population has become highly complex. Notably, the primary source of immunity has gradually shifted from natural infection to vaccination across generations, from older to younger individuals [3]. However, the recently reported high proportion of breakthrough infections in vaccinated age groups in recent years in China (22.2–75.1%) [4,5]—compared to age groups that previously experienced natural measles infections has created a challenging outlook for measles control and elimination. This challenge has been further exacerbated by the disruption of routine vaccination during the COVID-19 pandemic worldwide, including in China [6]. Given the critical role of population immunity in shaping the course of the measles epidemic [3], identifying immunity differences among populations with varying immunity backgrounds is necessary for both identifying measles immunity gaps and informing vaccination policies [7].

A population-based serological study can thus be conducted to explore how measles-specific immunity dynamics differ between populations with varying immunity backgrounds, particularly those with only vaccination or natural infection immunity. Previous studies on acute and patients with convalescent measles have described B-cell responses and antibody patterns in the United States in 1999 [8]. However, these studies did not examine the long-term dynamics of naturally acquired antibodies relative to the pre-defined protective threshold in the current Chinese population. The relationship between naturally acquired immunity levels and virus circulation has also not been well addressed, primarily due to methodological challenges in reconstructing individual immunity by birth. Disentangling immunity resulting from single or repeated exposure to the measles virus remains difficult. Moreover, while it is known that vaccine-induced immunity may confer lower response levels and faster-waning rates compared to natural infection, as observed with other pathogens [9,10], the extent to which peak immunity levels and decay parameters differ between routine vaccination and natural infection remains unclear in the Chinese population. With improved measles control in China following SIAs and a reduced risk of measles virus exposure, the absence of natural boosting may contribute to the waning of vaccine-induced immunity. However, no study has yet investigated this effect or the long-term antibody dynamics in Chinese children born in a near-elimination setting. These research gaps further restrict the development of epidemiological models used to project the potential for measles resurgence and assess the appropriateness of vaccination policy targeting infants born to women with complex immunological backgrounds today.

In the current study, we based our research on two cohort studies and one cross-sectional study, focusing on children who followed the current routine measles vaccination schedule and adults who acquired immunity through natural infection. We constructed a comprehensive database integrating vaccination, serology, and epidemic data to compare the dynamics of vaccine-induced and naturally acquired antibodies, analyze vaccine-induced antibody dynamics across birth cohorts, and investigate the factors influencing measles immunity in populations with distinct immune backgrounds.

## 2. Materials and Methods

### 2.1. Study Population and Data Collection

**Figure 1:**
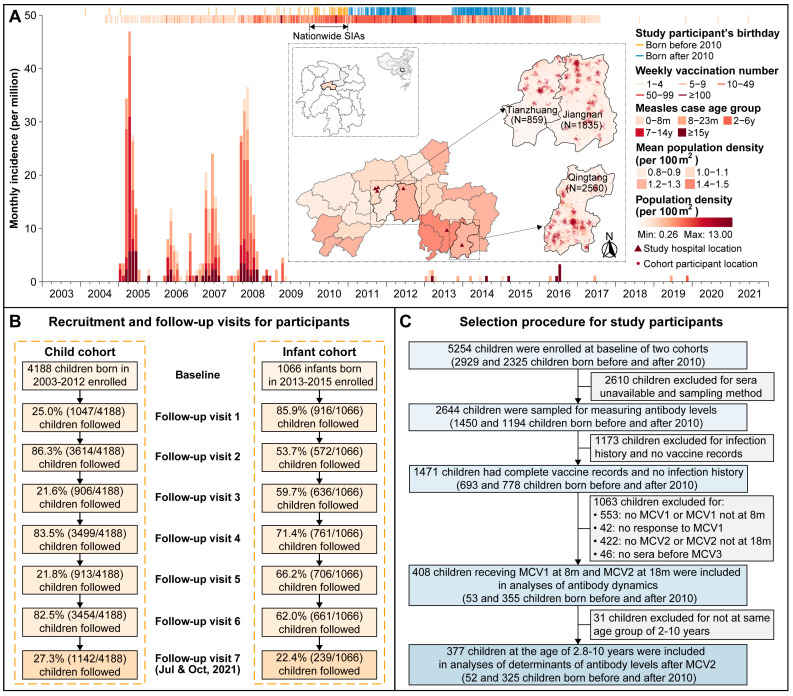
Selection procedure for study participants. (**A**) The geographic location of Anhua County, Yiyang City, Hunan Province, is displayed on the map. The density of the red color in each township represents the mean population density per 100 m^2^. Red points in the three townships (Tianzhuang, Qingtang, and Jiangnan) indicate the locations of cohort participants, while red triangles mark the six hospitals where infant cohort recruitment was conducted. A bar plot illustrates the monthly age-specific measles incidence in Anhua from 2003 to 2021. Weekly vaccination numbers for cohort participants with available vaccination records are shown in the red rug plot at the top of the panel. The birthdays of included study children, categorized by birth cohorts, are represented in the yellow and blue rugs below. (**B**) Recruitment and follow-up visits for all participants of two longitudinal cohorts. (**C**) Selection procedure for study participants.

The current study was based on previously established population-based cohort and cross-sectional studies conducted in three townships of Anhua County, Hunan Province, China (Figure 1A). The current study procedures have been described previously [11,12]. In brief, 4188 children aged 1–9 years were enrolled through stratified random sampling based on an age-specific registry list and were assessed six times at approximately six-month intervals between 2013 and 2015 (referred to as the “child cohort”). Additionally, 1066 mother-infant pairs were enrolled at delivery in six local hospitals, and the infants were followed six times (2, 4, 6, 12, 24, and 36 months) between 2015 and 2018 (referred to as the “infant cohort”). In July and October 2021, children from both cohorts who had attended at least four visits were invited to participate in an additional follow-up (Figure 1B). During the same period in 2021, a cross-sectional study was conducted, enrolling 1317 individuals aged 0–95 years through random selection from a complete household list of the selected townships. The proposed study population comprised participants from two cohort studies and one cross-sectional study. Eligible cohort children were included in the analysis of vaccine-induced antibodies, while eligible cross-sectional participants were included in the analysis of naturally acquired antibodies.

At each visit, a venous blood sample of 2 mL was drawn from cohort children, and 5 mL was collected from adults. Additionally, a 2 mL cord blood sample was drawn from infants at delivery. Socio-demographic information—including sex, birth date, annual household income, and the mother’s education level and occupation—along with delivery conditions (mode of delivery, term of pregnancy, and birthweight) and breastfeeding history were obtained from cohort participants. For cross-sectional participants, socio-demographic information (sex, birth date, income, education level, and occupation) was obtained. Vaccination cards, the official immunization documents in Mainland China, were collected from cohort children during the last three visits in 2016–2018 and again in 2021. Vaccination records were manually extracted and double-entered in *Microsoft Excel* by the authors. During the study period, the routine schedule had two minor modifications. During 2003−2005, the routine MCV consisted of two doses of monovalent measles vaccine (MV), administered at 8 months and 7 years of age, respectively. In 2005, the age at MCV2 was changed from 7 years to 18−24 months. In 2008, the second adjustment was implemented, modifying the vaccine types by stipulating that MCV1 should use measles-rubella combined vaccine (MR) and MCV2 should use measles-mumps-rubella combined vaccine (MMR), with the vaccination schedule unchanged (8 months and 18−24 months of age). A measles case surveillance form was obtained through the Measles Surveillance System to identify infected cohort participants and describe local measles outbreaks. Local measles outbreaks were defined according to the China National Measles Surveillance Guideline, which specifies the following criteria: (1) over two cases occur in a village within 10 days; (2) over five cases occur in a township within 10 days; or (3) the weekly measles incidence in a county exceeds the average incidence of the same period over the past five years by more than 100%. Population size data were extracted from the Annual Statistic Book of Yiyang City, and population density at 10 m × 10 m grid resolution was obtained from *WorldPop*.

In the current study, cohort participants were included in vaccine-induced antibody analysis only if they: (1) received two doses of MCVs at 8 and 18 months of age; (2) showed seroconversion following MCV1, defined as either a change from negative to positive or a four-fold increase in antibody levels before and after MCV1 [12,13]; (3) had no history of measles infection. To assess the effect of vaccination policy and the corresponding measles epidemic levels on individual antibody dynamics, eligible cohort children were further classified by their birth year (born before or after 2010). This classification was based on the fact that SIAs were rare after 2010, measles epidemic levels were low, and both groups exhibited sufficient sample sizes for analysis (Figure 1A and Figure S2A). Cross-sectional participants were included in the naturally acquired antibody analysis and were considered eligible if they were born before 1968 when the measles vaccine was introduced in Anhua County.

### 2.2. Laboratory Procedures

Given the availability of residual serum samples, 2629 children from original cohorts (50% of the total; 555 [52.1%] from the infant cohort and 2074 (49.5%) from the child cohort) and 1015 participants from the cross-sectional study were tested. This sample size met the minimum requirement for generalized additive mixed model (GAMM) and Bayesian modeling after the selection procedure [12,13]. Anti-measles IgG antibodies were tested using commercial enzyme-linked immunosorbent assay (ELISA) kits (SERION ELISA classic measles virus IgG; Institut Virion/Serion GmbH, Wurzburg, Germany) following the manufacturer’s instructions. Details of the sampling methods and ELISA procedures have been described elsewhere [12,13]. In brief, serum samples were diluted 1:100, added on the 96-well plate, and tested in parallel with ready-to-use negative control and standard sera. Optical density values of antibody levels were converted into international units (mIU/mL) using a calibration curve generated from the standard serum with *SERION32*. ELISA results were previously validated, demonstrating high consistency (95.8%) with the reduced plague neutralization test [13]. In the current study, we adopted the commonly accepted protective threshold of 200 mIU/mL [14,15].

### 2.3. Statistical Analyses

We first described the basic characteristics of all participants, and differences between groups were assessed using Wilcoxon rank-sum tests or chi-squared test/Fisher exact tests. Age-specific antibody levels were described using the geometric mean concentration with a 95% confidence interval (CI). All tests were two-sided, and *p*-values less than 0.05 were statistically significant. The dynamics of vaccine-induced antibodies were characterized using a previously established GAMM [13]. A linear mixed model was used to explore the association between antibody levels after two doses of MCVs and various factors, including birth cohort, sex, time since MCV2, mode of delivery, age at MCV1 and MCV2, initial antibody level, decay rate, and prior outbreak experience [12,13]. This model was expressed as follows:

(1) $$\begin{array}{cc} y_{i,j} & =E\left(y_{i,j}|x_{i,j},\;u_{i}\right)+\varepsilon_{i,j}\\ E\left(y_{i,j}|x_{i,j},\;u_{i}\right) & =\beta_{0}\left(\tau \right)+\beta_{1}\left(\tau \right)t_{i,j}+\beta_{2}\left(\tau \right)sex_{i}+\beta_{3}\left(\tau \right){delivery}_{i}+\beta_{4}\left(\tau \right){cohort}_{i}+u_{i} \end{array}$$

where $y_{ij}$ represents the log-transformed antibody level of individual i measured in j-th visit, and $E\left(y_{i,j}|x_{i,j},\;u_{i}\right)$ denotes the τ-th quantile of y conditioned on $x_{i,j}$ and $u_{i.}$ The term $\varepsilon_{ij}$ represents the independent error term, whereas $u_{i}$ is the individual random effect, and $x_{i,j}$ is the design matrix of predictors. The coefficient $\beta_{k}\left(\tau \right)$ (k: 1,2,3,4) measured the impact on τ-th quantile of antibody level. The variables $t_{i,j}$, $sex_{i}$, ${delivery}_{i}$, and ${cohort}_{i}$, refer to time since MCV2, sex, mode of delivery, and birth cohort.

Additionally, linear conditional quantile mixed models were constructed at three quantile levels (τ): 0.25, 0.50, and 0.75 to capture the effect across the entire distribution of antibody levels. For a given τ (0<τ<1), the model was expressed as follows:

(2) $$\begin{array}{cc} y_{i,j} & =Q_{y_{i,j}|u_{i}}\left(\tau |x_{i,j},\;u_{i}\right)+\varepsilon_{i,j}\left(\tau \right)\\ Q_{y_{i,j}|u_{i}}\left(\tau |x_{i,j},\;u_{i}\right) & =\beta_{0}\left(\tau \right)+\beta_{1}\left(\tau \right)t_{i,j}+\beta_{2}\left(\tau \right)sex_{i}+\beta_{3}\left(\tau \right){delivery}_{i}+\beta_{4}\left(\tau \right){cohort}_{i}+u_{i} \end{array}$$

where $Q_{y_{i,j}|u_{i}}\left(\tau |x_{i,j},\;u_{i}\right)$ and $\varepsilon_{ij}\left(\tau \right)$ represents the τ-th quantile of y and independent error term, conditioned on $x_{i,j}$ and $u_{i}$, respectively.

The optimal model was selected using the analysis of variance (ANOVA) test and goodness-of-fit statistics, including the Akaike information criterion (AIC), Bayesian information criterion (BIC), log-likelihood value, and explained variance based on manual stepwise regression. Parameters were estimated using the likelihood approach, and the parametric bootstrapping method was used to generate 95% CIs and *p*-values. In addition, we adopted an exponential model [12] of log-transformed antibody level to estimate the exponential decay rate of vaccine-induced antibodies.

To characterize the dynamics of naturally acquired antibodies, we developed a mechanistic model to determine birth cohort-specific antibody dynamics resulting from natural infections [12,16]. Given the high transmissibility of measles virus and long-lasting nature of infection-induced immunity [1], we assumed near-universal infection for participants born before measles vaccine introduction, with each individual experiencing only one infection in their lifetime. Assuming constant force of infection λ, the probability of infection at age t was $\lambda e^{-\lambda t}$ under the catalytic structure. Under the assumption of exponential decay of naturally acquired antibody, the observed antibody level for individual i at age a was given by:

(3) $$A_{0,t}e^{-\gamma (a-t)}$$

where $A_{0,t}$ represented the peak antibody level at the age of infection, and γ was the exponential decay rate. Furthermore, we modeled the overall antibody level at age a $\left(A_{i,a}\right)$ by integrating the antibody decay dynamics and infection probability:

(4) $$A_{i,a}=\int_{0}^{a}{\lambda e}^{-\lambda t}\times \left(A_{0,t}e^{-\gamma \left(a-t\right)}\right)dt$$

The prior distributions of parameters were obtained from published articles [8,12]. The model was implemented within a Bayesian framework, using a Hamiltonian Monte Carlo algorithm to obtain posterior estimates. Four independent chains were used, with each model having 1000 warmup samples and 3000 post-warm-up samples. Model convergence was assessed through visual assessment of chain mixing in trace plot, R-hat statistics, and effective sample size. The median of the posterior distribution for each parameter, along with the corresponding 95% credible interval (CrI), was calculated to obtain parameter estimates. The 95% CrI was defined as the 2.5th and 97.5th percentiles of the posterior distributions.

To explore the relationship between measles circulation in each year (measured as the annual incidence rate) and year-specific antibody levels, we performed wavelet analysis [17,18]. Before the transformation, the normalized data were extended using zero padding to a total length of $2\times 2^{n}$ (a total of 64 records) to reduce border effects. The final results were then truncated after the transformation. Continuous wavelet transformation was conducted to explore periodicity using the Morlet function as the mother wavelet. Based on a well-defined period identified from the power spectrum, wavelet reconstruction was used to analyze the wavelet phase angle (ranging from −180° to 180°) and describe the temporal relationship between year-specific measles incidence and antibody levels.

All analyses were conducted in R (version 4.2.3) and CmdStanR (version 1.4.0).

## 3. Results

### 3.1. Basic Characteristics of Study Participants

Of 5254 cohort children, 408 who received MCV according to routine vaccination schedule and met the inclusion criteria were included in the current study (Figure 1). Among them, 53 were born between 2004 and 2010 (referred to as children “born before 2010”), and 355 children between 2011 and 2015 (referred to as children “born after 2010”). The median age at baseline was 3.3 years (interquartile range [IQR]: 3.0–3.9) for children born before 2010 and 0 months (IQR: 0–1.5) for those born after 2010. The age at MCV1 did not differ between the two groups, with a median age of 8.3 months (IQR: 8.1–8.5) for children born before 2010 and 8.2 months (IQR: 8.0–8.6) for those born after 2010 (*p* = 0.683). Despite the modification of vaccine policy, the administered vaccine types remained consistent in two birth cohorts in the immunization practice. The predominant vaccine types for both MCV1 and MCV2 across birth cohorts were MR (97.3%) and MMR (96.6%), respectively. Age at MCV2 also did not differ between the two birth cohorts (before versus after 2010: 18.1 months [IQR: 18.0–18.5] and 18.2 months [IQR: 18.0–18.6], respectively; *p* = 0.315). The two birth cohorts were comparable in all other baseline characteristics (Table 1). Of the 1015 cross-sectional participants, 222 adults born between 1934 and 1967, aged from 54 to 84 years (median age: 66.8 years; IQR: 59.6–71.5) were included in the current study. Of these, 56.3% (*n* = 125) were females.

### 3.2. Vaccine-Induced Antibody

**Figure 2:**
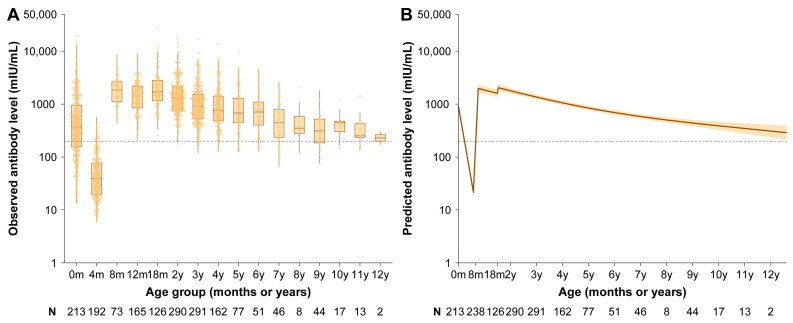
Measles antibody dynamics in study children. (A) Observed measles IgG antibody level (mIU/mL). (**B**) Predicted measles IgG antibody level (mIU/mL) based on the GAMM model. Box plots in (**A**) display the median (middle hinge), 25th and 75th quartiles (lower and upper hinges), and ± 1.5 times the interquartile range (lower and upper whiskers) of log-transformed measles IgG antibody levels. Points in (**A**) represent individual observed antibody levels. In (**B**), the solid line and orange shading indicate the mean of log-transformed antibody levels and the 95% CI predicted by GAMM. The horizontal dashed lines refer to the protective threshold of 200 mIU/mL. Numbers below the axes indicate the number of children in each age group and the sample size used to derive the CI.

The dynamics of the maternal and vaccine-induced antibodies of children following routine vaccination at 8 and 18 months of age are presented in Figure 2. We observed that maternal antibody levels declined rapidly after birth and were estimated to fall below the protective threshold at 2.8 months of age. After receiving the two-dose schedule at 8 and 18 months, measles antibody increased, peaking at 1987.4 mIU/mL (95% CI: 1690.8–2335.9) at the age of 9 months and 2011.5 mIU/mL (95% CI: 1746.7–2316.6) at 19 months. After MCV2, vaccine-induced antibodies declined steadily and were estimated to be 309.2 mIU/mL (95% CI: 235.0–406.9) at 12 years of age. Notably, children born after 2010 exhibited lower age-specific antibody levels between 6 and 10 years of age (Figure S2B). After adjusting for delivery mode and time since MCV2, being male and being born after 2010 were associated with lower antibody levels. Males exhibited antibody levels that were 21.3% lower (95% CI: 6.5–33.8%) compared to females, and children born after 2010 exhibited levels that were 21.4% lower (95% CI: 1.2–37.0%) compared to those born before 2010. The negative effect of birth year was more pronounced at the 25th quantile, where children born after 2010 exhibited 24.7% lower antibody levels (95% CI: 1.9–41.6%) compared to those born before 2010 (Table 2).

**Figure 3:**
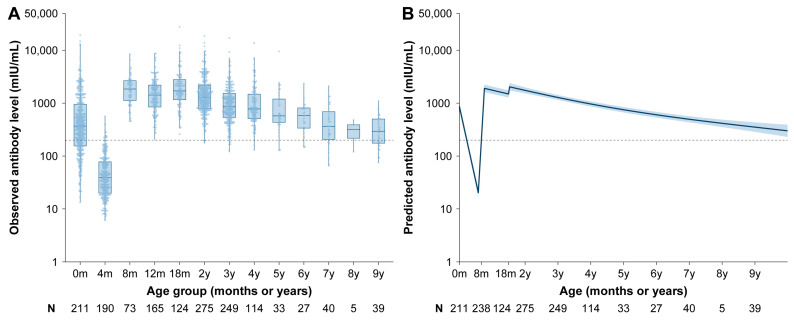
Measles antibody dynamics in children born after 2010 receiving routine vaccination schedule. (**A**) Observed measles IgG antibody level (mIU/mL). (**B**) Predicted measles IgG antibody level (mIU/mL) based on the GAMM model. Box plots in (**A**) display the median (middle hinge), 25th and 75th quartiles (lower and upper hinges), and ± 1.5 times the interquartile range (lower and upper whiskers) of log-transformed measles IgG antibody levels. Points in (**A**) represent individual observed antibody levels. In (**B**), the solid line and orange shading indicate the mean of log-transformed antibody levels and the 95% CI predicted by GAMM. The horizontal dashed lines refer to the protective threshold of 200 mIU/mL. Numbers below the axes indicate the number of children in each age group and the sample size used to derive the CI.

To further investigate the effect of the birth cohort on long-term vaccine-induced antibody dynamics, we analyzed the antibody trends in children born before and after 2010. While antibody dynamics were similar before MCV2 vaccination, post-MCV2 antibody levels declined more rapidly in children born after 2010 compared to those born before 2010, reaching 299.7 mIU/mL (95% CI: 230.2–390.3) by 10 years of age (Figure 3). The estimated age at which antibody levels fell below the protective threshold was 12.5 years for children born after 2010, compared to 15.8 years for all children (Figure S4). The estimated antibody decay rates were 3.4 × 10^−3^ log-log mIU/mL per year (95% CI: 3.6 × 10^−3^−3.2 × 10^−3^) for children born after 2010 and 3.0 × 10^−3^ log-log mIU/mL per year (95% CI: 3.2 × 10^−3^−2.9 × 10^−3^) for all children.

### 3.3. Naturally Acquired Antibody

**Figure 4:**
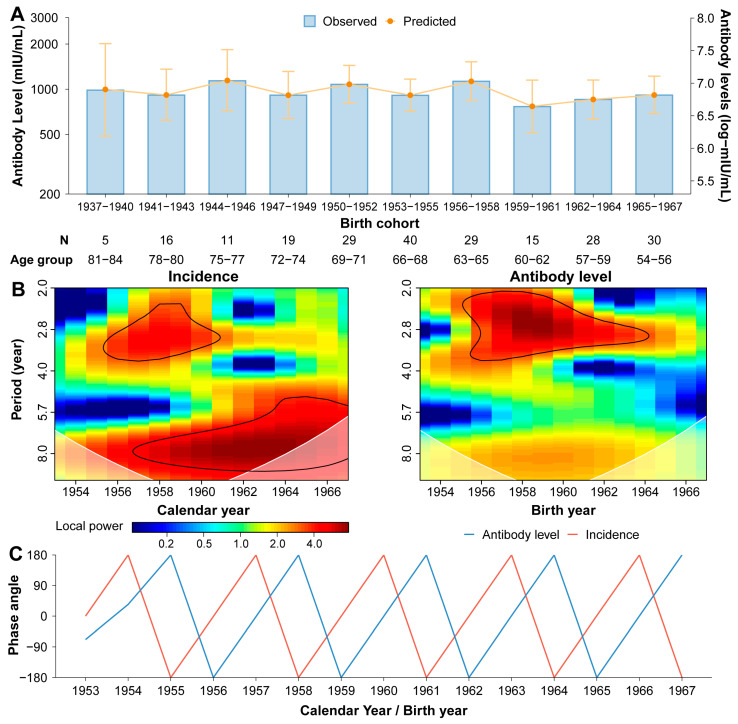
Naturally acquired antibody levels in relation to measles epidemics. (**A**) Observed and predicted age-specific antibody levels by birth cohorts. Orange points and error bars are the estimated age-specific measles antibody levels. (**B**) Wavelet power spectrum of the measles incidence from 1953 to 1967 and observed measles antibody levels in participants born during this period. White transparent polygons indicate the cone of influence, and black lines represent the 95% confidence levels computed from 1000 bootstrapped series. (**C**) Phase angle for the reconstructed signal, restricted to 3 years.

Antibody levels in the naturally infected population ranged from 913.0 mIU/mL (95% CI: 654.4–1273.8) to 1138.4 mIU/mL (95% CI: 741.9–1746.8) in individuals aged 54 to 84 years. The sampled posterior distributions were effectively explored, with all parameters demonstrating convergence and all chains indicating good mixing (Figure S7). The model accurately reproduced the observed serological patterns across all age groups, with fitted antibody levels ranging from 910.4 mIU/mL (95% CrI: 633.6–1309.2) and 1128.4 mIU/mL (95% CrI: 839.6–1515.8) (Figure 4A). The estimated force of infection (λ) was 0.30 (95% CrI: 0.21–0.40) per year. The exponential decay rate of naturally acquired antibodies was estimated at 2.30 × 10^−5^ log-log mIU/mL per year (95% CrI: 1.33 × 10^−5^−3.29 × 10^−5^) (Table S4), indicating a substantial difference from the decay rate of vaccine-induced antibodies. Peak antibody levels following natural infection varied across different birth cohorts, ranging from 854.1 mIU/mL (95% CrI: 639.1–1152.9) to 1152.9 mIU/mL (95% CrI: 713.4–1844.6), presenting a similar peak antibody level to vaccine-induced immunity (Table S4).

Wavelet transformation results revealed a similarity between long-term naturally acquired antibody levels in the population and the local measles incidence in their birth year. Similar period patterns were observed in both antibody levels and measles incidence, characterized by a short 3-year cycle and a longer 8-year cycle (Figure 4B). Analysis of the short period (3-year cycle) revealed that the phase angle, representing the timing of oscillation of the two signals, consistently demonstrated that measles incidence preceded antibody level throughout the study period (1953–1967). Specifically, a peak in measles incidence was followed by a corresponding peak in antibody levels after a one-year delay, a pattern that persisted throughout the study period (Figure 4C).

## 4. Discussion

In the current study, we observed the substantial difference in dynamics of naturally acquired and vaccine-induced antibodies, with higher long-term antibody levels following infection. As the vaccinated population constitutes the majority of current immunity, we further examined the long-term dynamics of vaccine-induced antibodies in Chinese children from different birth cohorts and found that children born after 2010 exhibited a faster antibody decay. Moreover, we identified sex and birth cohort as key factors influencing vaccine-induced antibodies and revealed a temporal relation between the epidemic level at birth year and long-term naturally acquired antibody levels.

The dynamics of vaccine-induced and naturally acquired antibodies were consistent with previous studies [8,13]. The vaccine-induced antibodies exhibited a decline trend after vaccination. The current study demonstrated that the protection provided by routine vaccine-induced antibodies lasted until age 15.8 years, which is longer than the previously reported 14.3 years under the same model structure [13]. This difference can be explained by multiphasic decay dynamics, characterized by a rapid drop followed by a slow decline [19]. The slow-decay phase is better characterized by an extended age span in the current study. Future studies can focus on characterizing the multiphase dynamics of measles antibodies. Additionally, the current study demonstrated the persistence and robustness of lifelong naturally acquired antibodies, which is consistent with previous studies using longitudinal sera [8]. In addition, with the catalytical model structure, we accounted for the uncertainty of infection timing and considered antibody decay from the point of infection, allowing for a longer decay assessment.

Different drivers of vaccine-induced and naturally acquired antibodies were investigated. Time since routine vaccination, male sex, and birth after 2010 were associated with lower long-term vaccine-induced antibody levels. Previous studies have also demonstrated sex differences in measles vaccine-related immunity, indicating that males tend to have a lower immune response to the measles vaccine and a faster decline in measles antibodies [20]. Children born after 2010, during a period of low measles incidence, exhibited lower mean and 25th quantile antibody levels. This relation suggests that measles epidemics play a vital role in maintaining vaccine-induced antibody levels, consistent with previous studies [21,22]. This effect can be explained by the secondary immune response (also known as “subclinical infection”) [23], in which circulating measles viruses boost antibody levels in exposed vaccines without causing clinical symptoms. This effect was more pronounced in vaccines with lower pre-exposure antibody levels [24].

The substantial difference in decay parameters between vaccine-induced and naturally acquired antibodies was identified using exponential decay models of log-transformed antibody levels [12]. The decay parameters for both vaccine-induced and naturally acquired antibodies are consistent with previous results [8,12,22]. Given the complexity of antibody decay dynamics [19], multiple parametric statistic models have been designed to describe antibody dynamics, with the most common being linear, exponential, and power-law models applied to log-transformed antibody levels [25]. The linear model is limited by its assumption of constant decay, which may lead to biased estimates of decay parameters [26]. A bi-phasic model has also been used to describe potential changes in decay patterns over time following vaccination or infection [27]. Future studies could integrate statistical and mechanistic models to elucidate the underlying decay mechanisms and reconstruct long-term antibody dynamics. While antibodies induced by vaccination waned faster than those from natural infection in our study, vaccination still provided durable and effective protection. High vaccine coverage remains essential to sustain herd immunity [1].

The current study faced several limitations. Loss to follow-up may introduce selection bias. However, we found that the follow-up rate of different quantiles (25th, 50th, and 75th) of initial antibody levels was comparable at the ages 6–10, indicating that the potential selection bias due to loss of follow-up is minimal. Current model primarily focused on the decay of antibodies following a one-time measles infection, while potential subclinical infections (which are relatively rare in naturally infected populations [28,29]) were not accounted for. The current study only measured humoral immunity, while T-cell-mediated immunity plays a crucial role in protecting against measles infection [30]. Due to data limitations, the current study could not investigate the effect of breastfeeding on long-term antibody levels [31], which may contribute to antibody persistence through mucosal immunity. Additionally, we were unable to describe the dynamics and determinants of antibody levels after MCV3 vaccination due to loss of follow-up. Future studies could explore the role and persistence of cellular and mucosal immunity under different vaccine schedules. Furthermore, because incidence data before vaccine introduction was reported annually, the precision of the wavelet analysis was limited. More detailed data could enhance future analysis.

## 5. Conclusions

In conclusion, our findings revealed immunity heterogeneity among individuals with different immune backgrounds. The faster decay of vaccine-induced antibodies in young generations born in settings with low measles incidence highlights generation-specific susceptibility to measles despite full vaccination. This suggests that population immunity levels should be actively reevaluated as measles incidence changes and that more attention should be given to young generations in future catch-up vaccination efforts.

## Figures and Tables

**Table 1:** Basic characteristics of study participants by birth cohort.

	Total (N = 408)	Born Before 2010 (N = 53)	Born After 2010 (N = 355)	*p*-Value
Age at baseline, years				
Median (Interquartile range, IQR)	0 (0−1.9)	3.28 (3−3.9)	0 (0−1.5)	4.13 × 10^−34^
Sex ^a^				
Male	199 (48.8)	23 (43.4)	176 (49.6)	0.488
Female	209 (51.2)	30 (56.6)	179 (50.4)	
Socioeconomic status ^b^				
Low	64 (15.7)	10 (18.9)	54 (15.2)	0.941
Middle	198 (48.5)	28 (52.8)	170 (47.9)	
High	98 (24.0)	15 (28.3)	83 (23.4)	
Missing	48 (11.8)	0 (0)	48 (13.5)	
Mode of delivery				
Vaginal delivery	255 (62.5)	35 (66.0)	220 (62.0)	0.676
Caesarean section	153 (37.5)	18 (34.0)	135 (38.0)	
Term of pregnancy				
Preterm birth	17 (4.2)	2 (3.8)	15 (4.2)	0.538
Full-term birth	379 (92.9)	51 (96.2)	328 (92.4)	
Post-term birth	12 (2.9)	0 (0.0)	12 (3.4)	
Birth weight, grams				
Median (IQR)	3300 (3000−3600)	3250 (2925−3500)	3300 (3000−3600)	0.509
Breastfeeding before 6 months				
No	35 (8.6)	6 (11.3)	29 (8.2)	0.432
Yes	373 (91.4)	47 (88.7)	326 (91.8)	
MCV doses received				
2 doses	403 (98.8)	51 (96.2)	352 (99.2)	0.128
3 doses	5 (1.2)	2 (3.8)	3 (0.8)	
Age at MCV1, months				
Median (IQR)	8.2 (8.0−8.5)	8.3 (8.1−8.5)	8.2 (8.0−8.6)	0.683
Age at MCV2, months				
Median (IQR)	18.2 (18.0−18.5)	18.1 (18.0−18.5)	18.2 (18.0−18.6)	0.315

^a^ Variables are revealed as *n* (%) unless otherwise specified. ^b^ Socioeconomic status is calculated based on annual household income, mother’s educational level, and mother’s occupation.

**Table 2:** Determinants of MCV2 antibody levels in children receiving two doses of MCV according to the routine vaccination schedule.

	Participants’ Characteristics	Mean		Q25 (τ = 0.25)		Q50 (τ = 0.50)		Q75 (τ = 0.75)	
		β (95% CI)	*p*-Value	β (95% CI)	*p*-Value	β (95% CI)	*p*-Value	β (95% CI)	*p*-Value
Intercept		7.29 (7.05, 7.53)	0.00	7.34 (7.03, 7.65)	1.35 × 10^−254^	7.38 (7.07, 7.68)	5.91 × 10^−260^	7.38 (7.08, 7.69)	2.66 × 10^−261^
Time since MCV2, months ^b^									
Median (IQR)	31.2 (21.3, 47.7)	−0.01 (−0.02, −0.01)	0.001	−0.02 (−0.02, −0.01)	4.20 × 10^−9^	−0.01 (−0.02, −0.01)	1.10 × 10^−8^	−0.01 (−0.02, −0.01)	4.48 × 10^−10^
Sex ^a^									
Male	180 (47.7)	Reference	−	Reference	−	Reference	−	Reference	−
Female	197 (52.3)	0.24 (0.07, 0.41)	0.006	0.12 (−0.07, 0.32)	0.215	0.14 (−0.05, 0.34)	0.146	0.16 (−0.03, 0.36)	0.097
Mode of delivery									
Vaginal delivery	236 (62.6)	Reference	−	Reference	−	Reference	−	Reference	−
Caesarean section	141 (37.4)	−0.06 (−0.23, 0.11)	0.411	−0.02 (−0.19, 0.16)	0.864	0.00 (−0.18, 0.17)	0.986	−0.02 (−0.19, 0.15)	0.828
Birth cohort									
Before 2010	52 (13.8)	Reference	−	Reference	−	Reference	−	Reference	−
After 2010	325 (86.2)	−0.23 (−0.46, −0.01)	0.046	−0.29 (−0.55, −0.02)	0.036	−0.25 (−0.52, 0.01)	0.061	−0.23 (−0.50, 0.04)	0.093

^a^ Time since MCV2 refers to the period when 883 serum samples were collected from 377 included children. ^b^ Variables are presented as *n* (%) unless otherwise specified.

## Data Availability

The data presented in this study are available on request from the corresponding author, as individual-level epidemiological and serological data contain sensitive information that cannot be publicly shared due to privacy and ethical restrictions.

## Supplementary Materials

The following supporting information can be downloaded at: https://www.mdpi.com/article/10.3390/vaccines13060555/s1.

## Abbreviations

The following abbreviations are used in this manuscript:

MCV	measles-containing vaccine
MV	monovalent measles vaccine
MR	measles-rubella combined vaccine
MMR	measles-mumps-rubella combined vaccine
SIA	supplementary immunization activity
ELISA	enzyme-linked immunosorbent assay
GAMM	generalized additive mixed model
CI	confidence interval
