# Supplementary material for: Comparative Analysis of Long-Term Measles Immune Response After Natural Infection and Routine Vaccination in China
Source: Vaccines (Basel). 2025 May 23;13(6):555. doi: 10.3390/vaccines13060555 (PMC12197537; doi:10.3390/vaccines13060555)
Supplement: Supplementary file 1 [file vaccines-13-00555-s001.zip › vaccines-3608452-supplementary.pdf]

Supplementary information

Tables

Table S1. Basic characteristics by birth cohort in children included in analyses of determinants of MCV2-antibody levels.

	Overall (N=377)	Before 2010 (N=52)	After 2010 (N=325)	P-value
**Initial age, years**[a]				
Median (IQR)	3.16 (3.0, 3.4)	3.28 (3.0, 3.9)	3.15 (3.0, 3.4)	**0.023**
**Sex**[b]				
Male	180 (47.7)	23 (44.2)	157 (48.3)	0.691
Female	197 (52.3)	29 (55.8)	168 (51.7)	
**Mode of delivery**				
Vaginal delivery	236 (62.6)	34 (65.4)	202 (62.2)	0.770
Caesarean section	141 (37.4)	18 (34.6)	123 (37.8)	
**Age at MCV1, months**				
Median (IQR)	8.2 (8.0, 8.6)	8.27 (8.0, 8.5)	8.2 (8.0, 8.6)	0.741
**Age at MCV2, months**				
Median (IQR)	18.23 (18.0, 18.5)	18.13 (18.0, 18.5)	18.23 (18.0, 18.6)	0.327
**Initial antibody level, log mIU/mL**[a]				
Median (IQR)	6.9 (6.3, 7.4)	7.15 (6.3, 7.4)	6.85 (6.3, 7.4)	0.230
**Mean decay rate, log mIU/mL per month**[c]				
Median (IQR)	-0.02 (-0.02, -0.01)	-0.01 (-0.02, -0.01)	-0.02 (-0.02, -0.01)	0.330
Missing (n; %)	142 (37.7)	2 (3.8)	140 (43.1)	
**Experienced outbreak**[d]				
No	368 (97.6)	43 (82.7)	325 (100)	**9.55e-09**
Yes	9 (2.4)	9 (17.3)	0 (0)	

[a] Initial age and initial antibody level refer to the age and according antibody level when children were first followed after receiving MCV2.

[b] Variables are shown as n (%) unless otherwise specified.

[c] Mean decay rate is defined as the difference of antibody level divided by time span. 142 children (2 born before 2010 and 140 born after 2010) with only one serum sample after MCV2 are not included in the calculation of decay rate.

[d] Experienced outbreak is defined as whether any local outbreak has occurred before a child's enrollment in cohorts.

Table S2. Univariable regression of determinants of MCV2-antibody levels in the linear mixed model and linear quantile models of Q25, Q50, and Q75.

	Characteristics	Mean		Q25 (τ=0.25)		Q50 (τ=0.50)		Q75 (τ=0.75)	
		β (95% CI)	P-value	β (95% CI)	P-value	β (95% CI)	P-value	β (95% CI)	P-value
**Time since MCV2, months**									
Median (IQR)	31.2 (21.3, 47.8)	-0.01 (-0.02, -0.01)	**1.46e-105**	-0.02 (-0.02, -0.01)	**4.88e-08**	-0.01 (-0.02, -0.01)	**1.47e-08**	-0.01 (-0.01, -0.01)	**1.15e-06**
**Sex[a]**									
Male	431 (48.8)	Reference	-	Reference	-	Reference	-	Reference	-
Female	452 (51.2)	0.26 (0.09, 0.42)	**0.002**	0.07 (-0.16, 0.29)	0.547	0.37 (0.08, 0.66)	**0.014**	0.08 (-0.15, 0.32)	0.471
**Mode of delivery**									
Vaginal delivery	556 (63.0)	Reference	-	Reference	-	Reference	-	Reference	-
Caesarean section	327 (37.0)	-0.05 (-0.22, 0.13)	0.601	-0.08 (-0.22, 0.07)	0.311	0.11 (-0.27, 0.49)	0.555	0.05 (-0.10, 0.19)	0.525
**Age at MCV1**									
Median (IQR)	8.23 (8.1, 8.6)	0.03 (-0.26, 0.31)	0.860	-0.13 (-0.44, 0.19)	0.422	-0.07 (-0.38, 0.24)	0.670	-0.03 (-0.39, 0.33)	0.849
**Age at MCV2**									
Median (IQR)	18.2 (18.0, 18.6)	-0.06 (-0.34, 0.22)	0.697	-0.05 (-0.42, 0.32)	0.803	-0.02 (-0.38, 0.33)	0.897	0.00 (-0.28, 0.28)	0.983
**Birth cohort**									
Before 2010	223 (25.3)	Reference	-	Reference	-	Reference	-	Reference	-
After 2010	660 (74.7)	-0.08 (-0.32, 0.15)	0.486	0.01 (-0.45, 0.47)	0.953	-0.20 (-0.70, 0.31)	0.438	-0.09 (-0.33, 0.16)	0.478
**Experienced outbreak**									
No	852 (96.5)	Reference	-	Reference	-	Reference	-	Reference	-
Yes	31 (3.5)	-0.14 (-0.67, 0.39)	0.608	-0.25 (-0.90, 0.39)	0.434	0.00 (-0.52, 0.52)	0.998	-0.17 (-0.77, 0.43)	0.570
**Initial antibody level[b]**									
Median (IQR)	7 (6.33, 7.4)	0.92 (0.89, 0.96)	**2.42e-167**	0.90 (0.85, 0.95)	**5.53e-36**	0.97 (0.92, 1.03)	**1.96e-37**	1.00 (0.96, 1.04)	**1.43e-45**
**Mean decay rate[b]**									
Median (IQR)	-0.02 (-0.03, 0)	3.65 (2.51, 4.78)	**1.20e-09**	5.23 (2.61, 7.85)	**2.05e-04**	5.22 (2.04, 8.40)	**0.002**	5.18 (2.49, 7.88)	**3.27e-04**

[a] Variables are shown as n (%) unless otherwise specified.

[b] Two variable were excluded from stepwise regression due to the linearity between them (FigureS6).

Table S3. Stepwise regressions of determinants of MCV2-antibody levels in the linear mixed model.

	Step1		Step2		Step3		Step4		Step5		Step6	
	**β (95% CI)**	**P-value**	**β (95% CI)**	**P-value**	**β (95% CI)**	**P-value**	**β (95% CI)**	**P-value**	**β (95% CI)**	**P-value**	**β (95% CI)**	**P-value**
**Intercept**	7.20 (7.11, 7.29)	**0.00e+00**	7.07 (6.95, 7.19)	**0.00e+00**	7.27 (7.04, 7.51)	**1.43e-198**	7.29 (7.05, 7.53)	**1.93e-195**	7.10 (4.86, 9.35)	**1.53e-09**	7.91 (2.72, 13.09)	**0.003**
**Time since MCV2**												
Median (IQR)	-0.01 (-0.02, -0.01)	**1.46e-105**	-0.01 (-0.02, -0.01)	**1.26e-105**	-0.01 (-0.02, -0.01)	**8.47e-106**	-0.01 (-0.02, -0.01)	**7.92e-106**	-0.01 (-0.02, -0.01)	**7.84e-106**	-0.01 (-0.02, -0.01)	**7.76e-106**
**Sex**												
Male			Reference	-	Reference	-	Reference	-	Reference	-	Reference	-
Female			0.25 (0.09, 0.41)	**0.003**	0.24 (0.08, 0.40)	**0.003**	0.24 (0.08, 0.40)	**0.003**	0.24 (0.08, 0.40)	**0.003**	0.24 (0.08, 0.40)	**0.003**
**Birth cohort**												
Before 2010					Reference	-	Reference	-	Reference	-	Reference	-
After 2010					-0.23 (-0.46, -0.01)	**0.045**	-0.23 (-0.46, 0.00)	**0.047**	-0.23 (-0.46, 0.00)	**0.047**	-0.23 (-0.46, 0.00)	**0.048**
**Mode of delivery**												
Vaginal delivery							Reference	-	Reference	-	Reference	-
Caesarean section							-0.06 (-0.23, 0.10)	0.454	-0.06 (-0.23, 0.10)	0.456	-0.06 (-0.23, 0.10)	0.469
**Age at MCV1**												
Median (IQR)									0.02 (-0.25, 0.29)	0.868	0.03 (-0.24, 0.30)	0.842
**Age at MCV2**												
Median (IQR)											-0.05 (-0.31, 0.22)	0.736
**Goodness of fit**												
**AIC (ΔAIC)**	1366		1362.3 (-3.7)		1362.7 (0.5)		1367.3 (4.5)		1371.4 (4.1)		1375.4 (4)	
**BIC**	1385.1		1386.2		1391.4		1400.7		1409.6		1418.4	
**Log-likelihood**	-679.0		-676.1		-675.4		-676.6		-677.7		-678.7	
**Explained variance (marginal)**	0.1340		0.1549		0.1571		0.1587		0.1585		0.1582	
**Explained variance (conditional)**	0.8928		0.8934		0.8928		0.8932		0.8934		0.8936	
χ^2	-		8.918		4.035		0.561		0.028		0.114	
**P-value[a]**	-		**0.003**		**0.045**		0.454		0.868		0.736	

[a]ANOVA tests are performed.

Table S4. Summary of model parameters, priors and posterior estimates for naturally-acquired antibody decay model.

Parameters	Prior distribution	Posterior estimates (median, 95% CrI)	Back-transformed result (median, 95% CrI)
**λ: Force of infection**	N(0.30, 0.005)	0.30 (0.21, 0.39)	-
**$A_{0,t}$: Naturally-acquired peak antibody level following infection for t-th birth cohort** [a]			
A_{01}: Peak antibody level following infection in 1937-1940 (0–3 years of age)	N(7.50, 3.00)	6.90 (6.21, 7.60)	992.27 (497.70, 1998.20)
A_{02}: Peak antibody level following infection in 1941-1943 (4–6 years of age)	N(7.50, 3.00)	6.82 (6.43, 7.22)	915.99 (620.17, 1366.49)
A_{03}: Peak antibody level following infection in 1944-1946 (7–9 years of age)	N(7.50, 3.00)	7.05 (6.57, 7.52)	1152.86 (713.37, 1844.57)
A_{04}: Peak antibody level following infection in 1947-1949 (10–12 years of age)	N(7.50, 3.00)	6.82 (6.45, 7.18)	915.99 (632.70, 1312.91)
A_{05}: Peak antibody level following infection in 1950-1952 (13–15 years of age)	N(7.50, 3.00)	6.98 (6.68, 7.27)	1074.92 (796.32, 1436.55)
A_{06}: Peak antibody level following infection in 1953-1955 (16–18 years of age)	N(7.50, 3.00)	6.81 (6.57, 7.06)	906.87 (713.37, 1164.45)
A_{07}: Peak antibody level following infection in 1956-1958 (19–21 years of age)	N(7.50, 3.00)	7.03 (6.75, 7.32)	1130.03 (854.06, 1510.20)
A_{08}: Peak antibody level following infection in 1959-1961 (22–24 years of age)	N(7.50, 3.00)	6.64 (6.23, 7.05)	765.09 (507.76, 1152.86)
A_{09}: Peak antibody level following infection in 1959-1961 (25–27 years of age)	N(7.50, 3.00)	6.75 (6.46, 7.05)	854.06 (639.06, 1152.86)
A_{010}: Peak antibody level following infection in 1959-1961 (28–30 years of age)	N(7.50, 3.00)	6.82 (6.53, 7.10)	915.99 (685.40, 1211.97)
**γ: Decay rate of naturally-acquired antibody level**	N(3e-03, 5e-05)	2.30e-05 (1.33e-05, 3.29e-05)	-
**σ_{obs}: Measurement error**	N(0, 1)	0.79 (0.72, 0.87)	-

[a] Equivalent to the age of getting infection for population born in 1937.

Figures

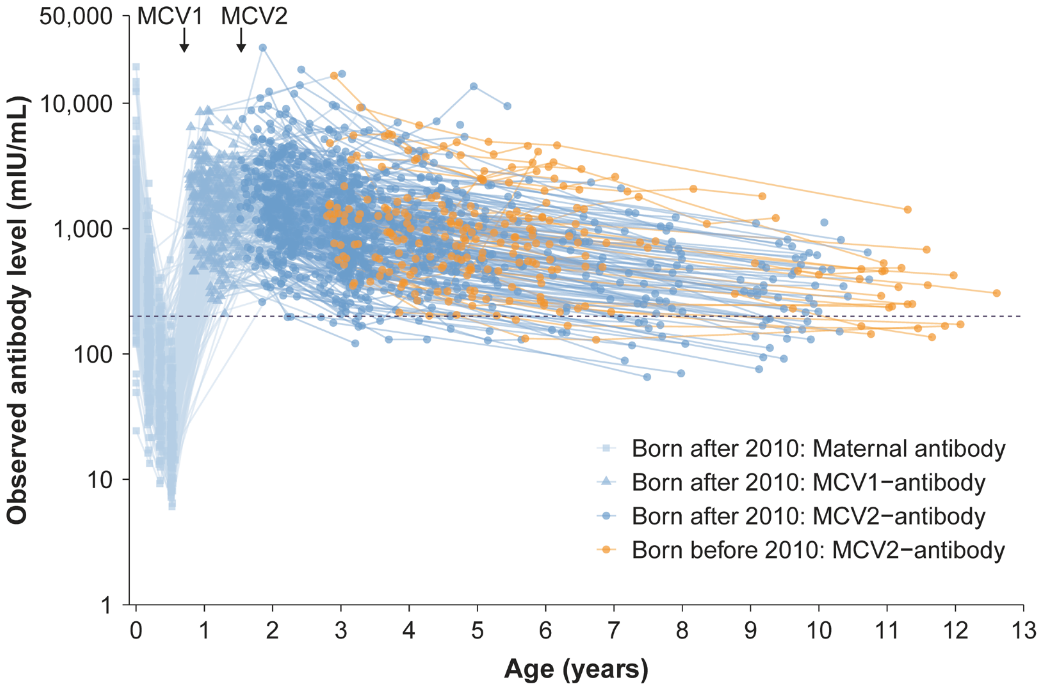

Figure S1. Individual antibody trajectory of children receiving two doses of MCVs in accordance with routine schedule. The horizontal dashed line refers to the protective threshold of 200 mIU/mL.

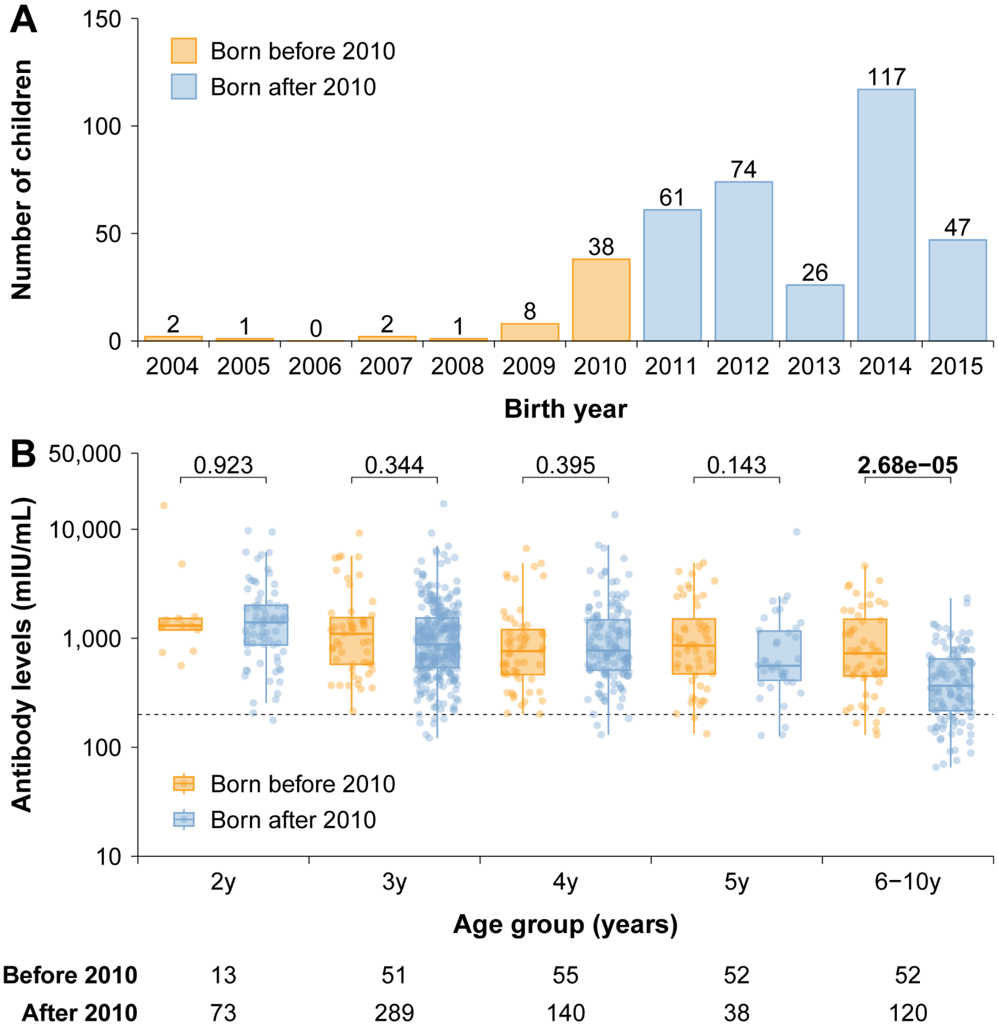

Figure S2. Division of birth cohort and age-specific MCV2-antibody levels by birth cohorts. The horizontal dashed line refers to the protective threshold of 200 mIU/mL.

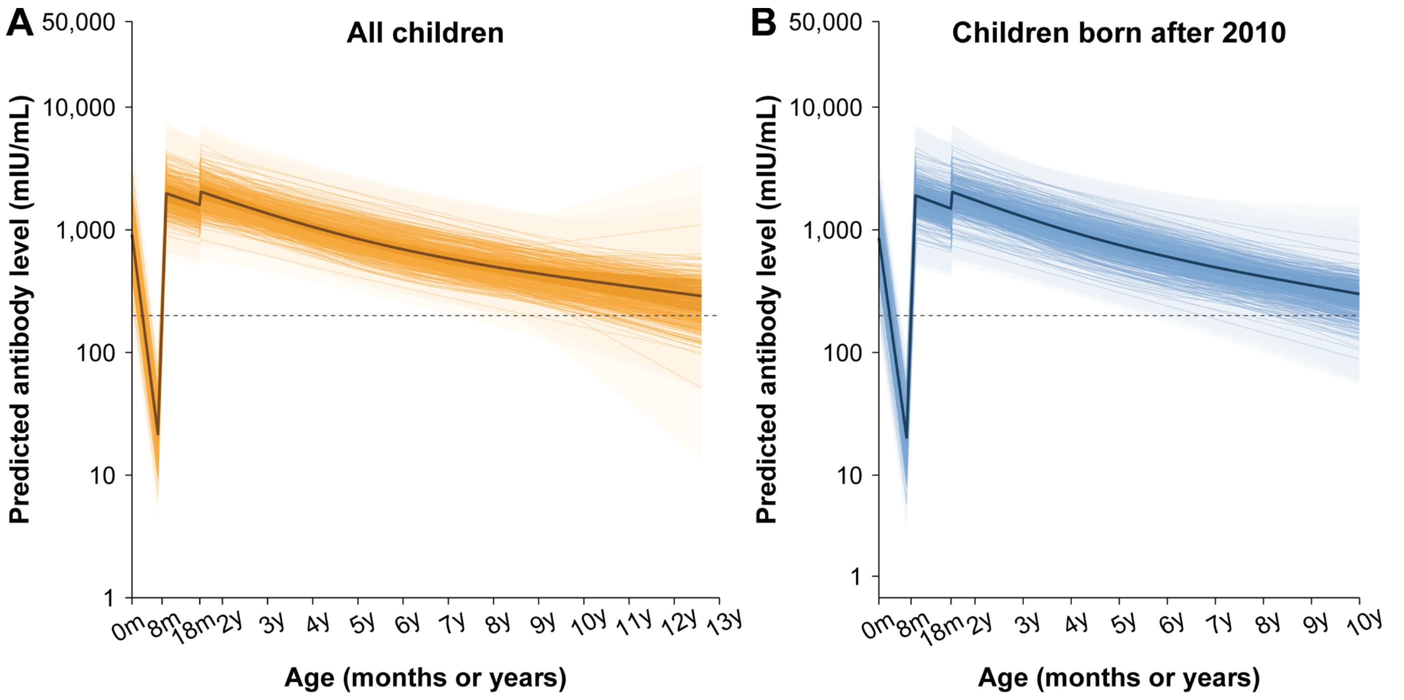

Figure S3. Measles antibody dynamics in children receiving two doses of MCV in accordance with routine vaccination schedule using observed sample and repeatedly drawn bootstrap samples. (A) Antibody dynamics in all included children. **(B)** Antibody dynamics in children born after 2010. The light orange/blue line are the fitted antibody levels of 500 replicates of bootstrap sample (sample size = 50) with 95% CIs shown as shaded ribbons. The brown and deep blue lines are the fitted antibody levels using the observed sample. The horizontal dashed lines refer to the protective threshold of 200 mIU/mL.

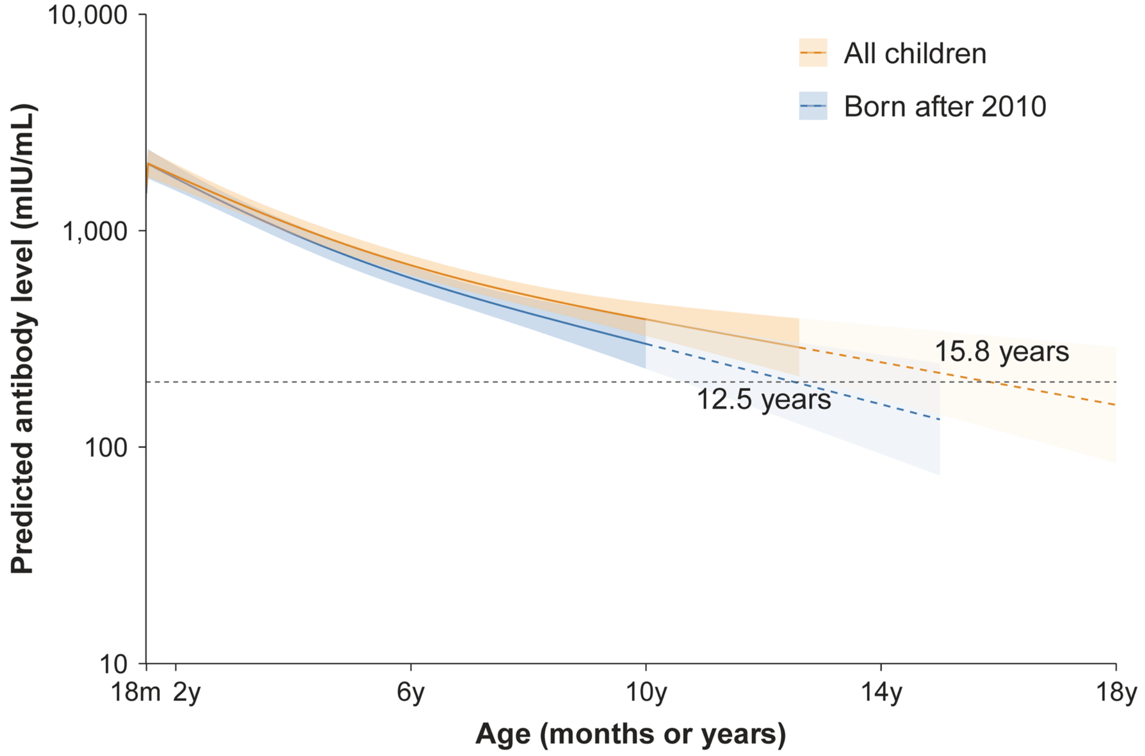

Figure S4. Predicted time to loss protection in children receiving 2 doses of MCV in accordance with routine schedule. The solid line and ribbon with the deeper color refer to the fitted values and according 95% confidence interval using observed dataset; the dashed line and ribbon with the lighter color refer to the extrapolated values and according 95% confidence interval based on GAMMs. The horizontal dashed line refers to the protective threshold of 200 mIU/mL.

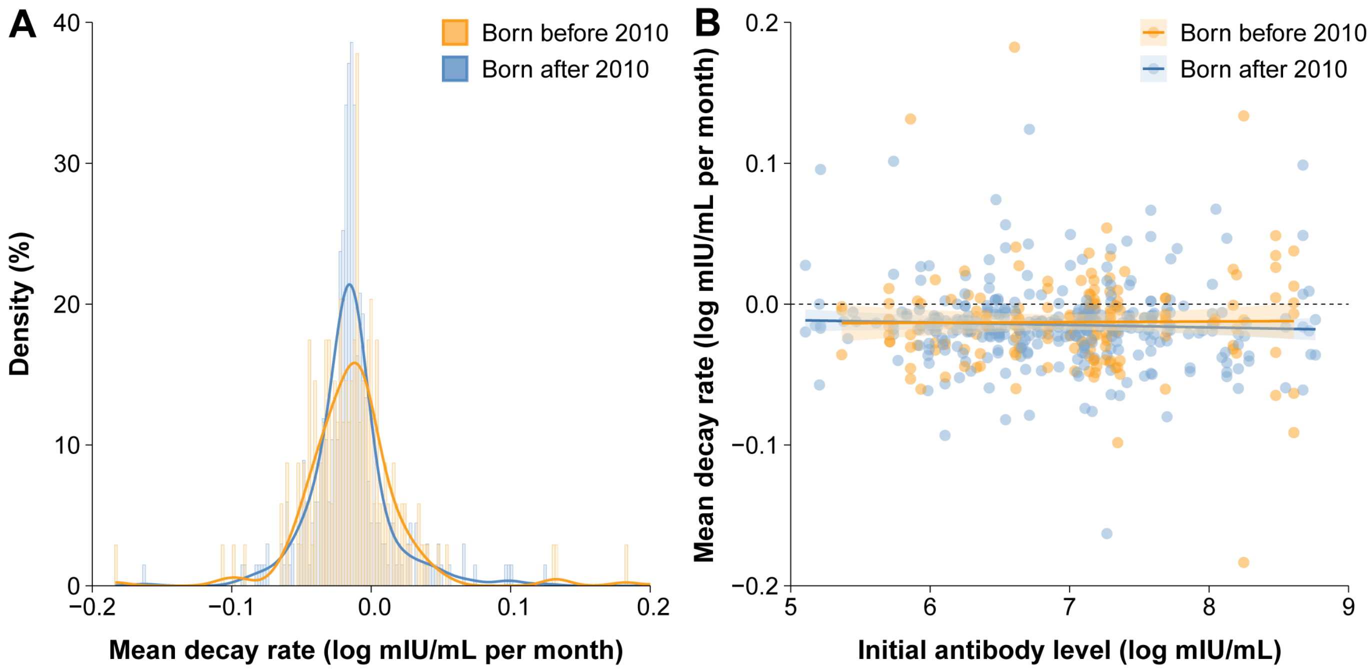

Figure S5. Distribution of antibody decay rate and the relation of decay rate and initial antibody level by birth cohort. Initial antibody refers to the antibody level when MCV2-antibody levels when it is first observed. P-values of Wald test for the linear regression of decay rate and initial antibody level: 0.045. Initial antibody level refers to the age and according antibody level when children were first followed after receiving MCV2. Mean decay rate is defined as the difference of antibody level divided by time span. 142 children (2 born before 2010 and 140 born after 2010) with only one serum sample after MCV2 are not included in the calculation of decay rate.

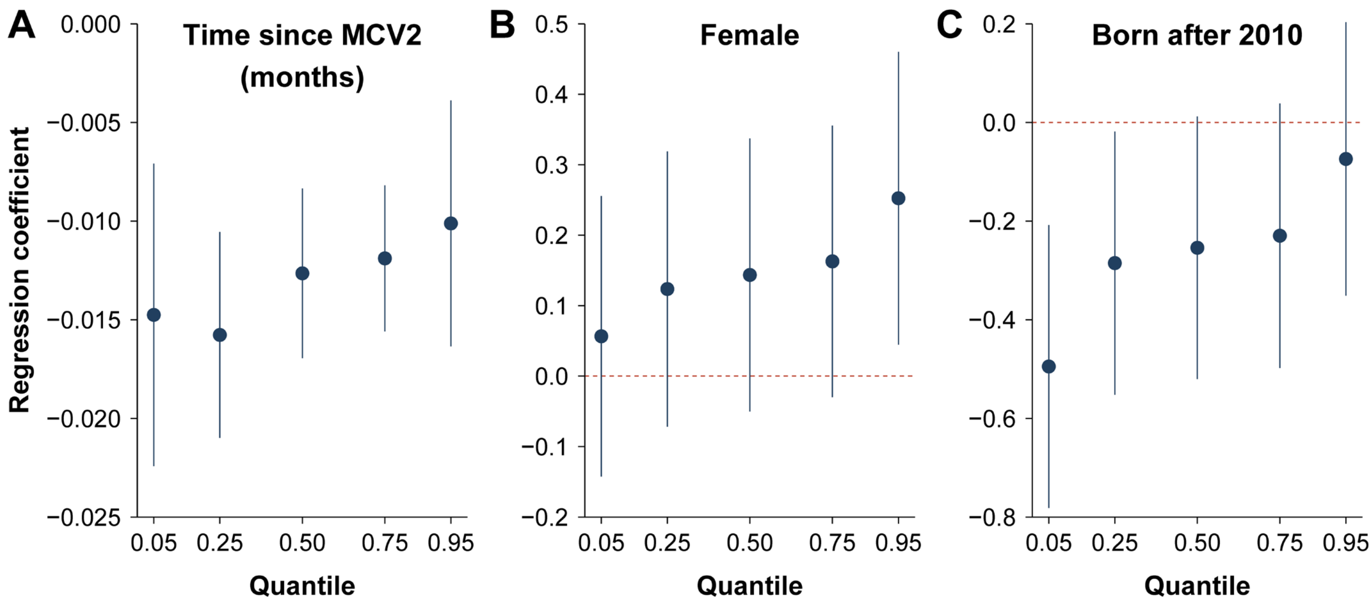

Figure S6. Regression coefficients in linear quantile mixed models. The blue points and vertical solid blue lines indicate estimated regression coefficients and 95% CIs at 5th, 25th, 50th, 75th and 95th quantiles.

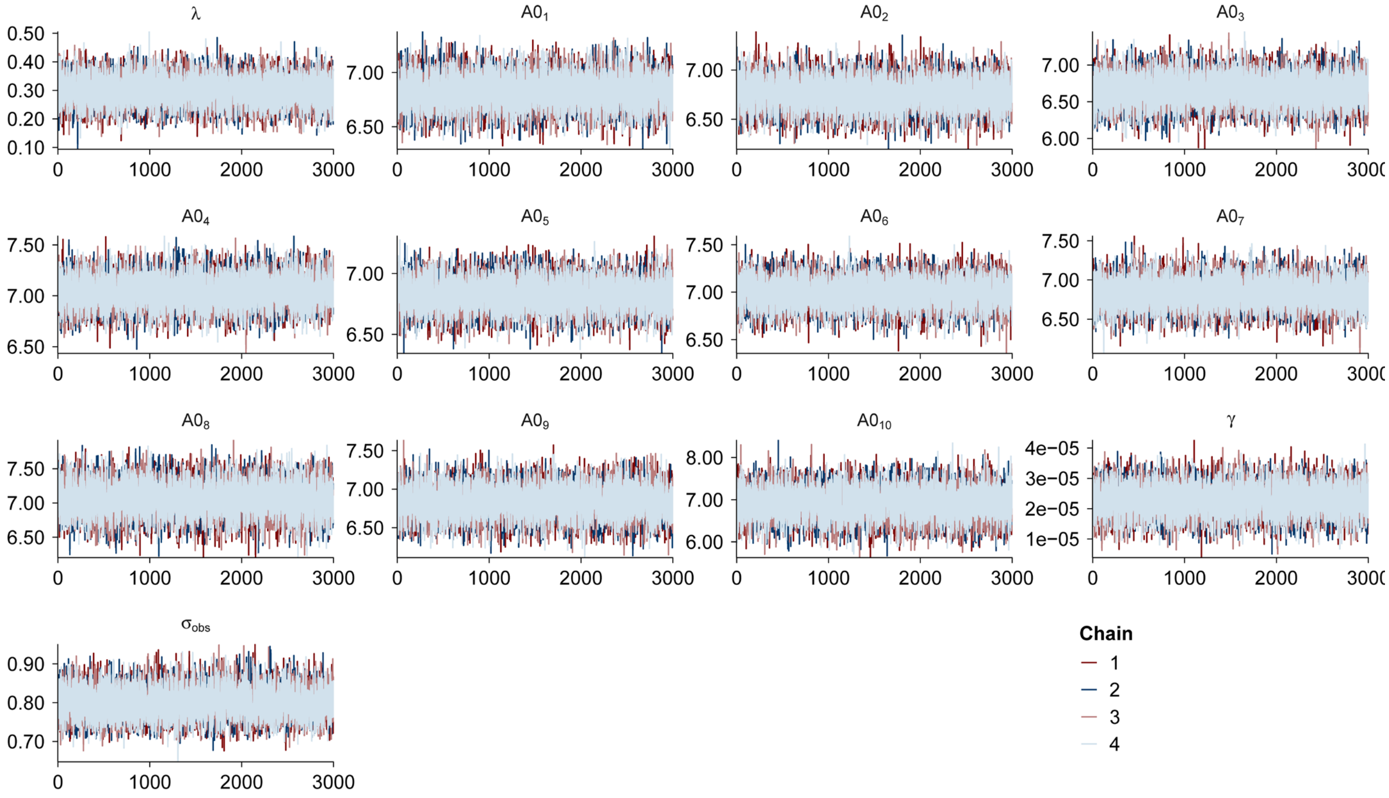

Figure S7. Trace plot of MCMC chains.
